# Supplementary material for: Design of Smart Nanodiamonds: Introducing pH Sensitivity to Improve Nucleic Acid Carrier Efficiency of Diamoplexes
Source: Pharmaceutics. 2022 Aug 26;14(9):1794. doi: 10.3390/pharmaceutics14091794 (PMC9501119; doi:10.3390/pharmaceutics14091794)
Supplement: Supplementary file 1 [file pharmaceutics-14-01794-s001.zip › pharmaceutics-1866664-supplementary.pdf]

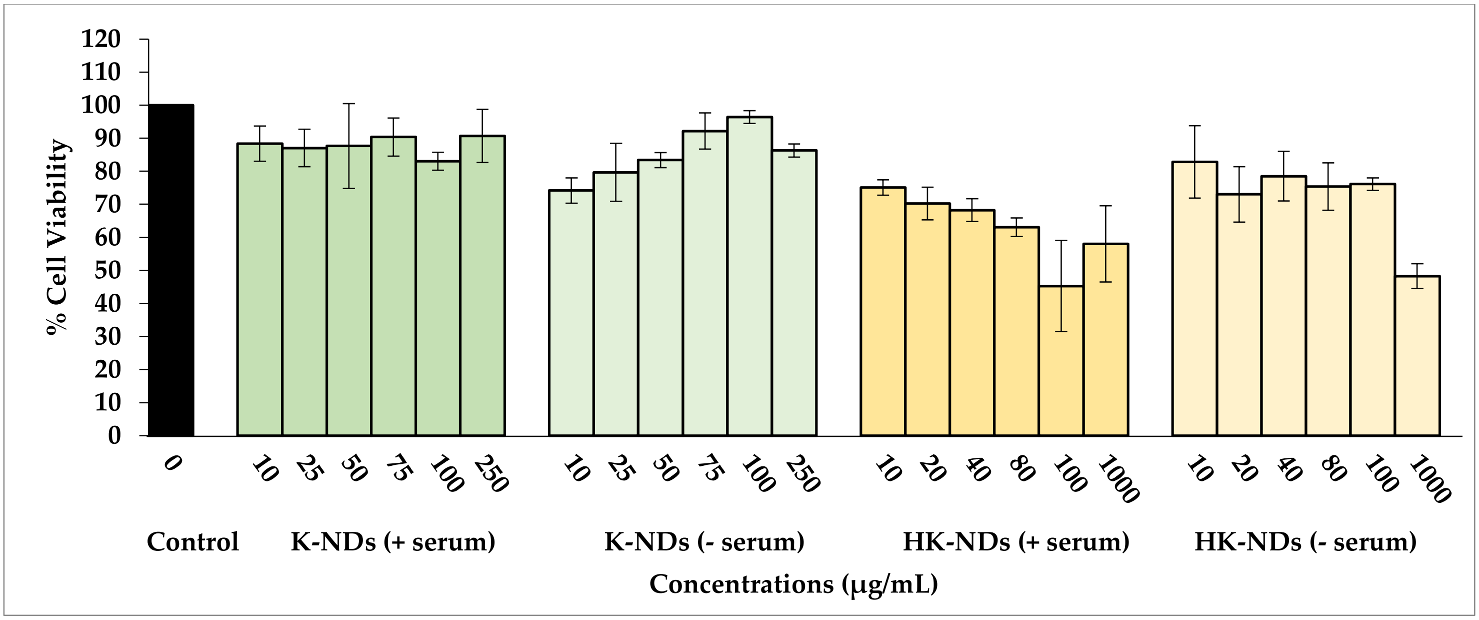

Figure S1. Comparison of biocompatibility profiles of K-NDs and HK-NDs in the presence and absence of serum proteins. Unlike K-NDs, HK-NDs generally are biocompatible in the absence of serum proteins.

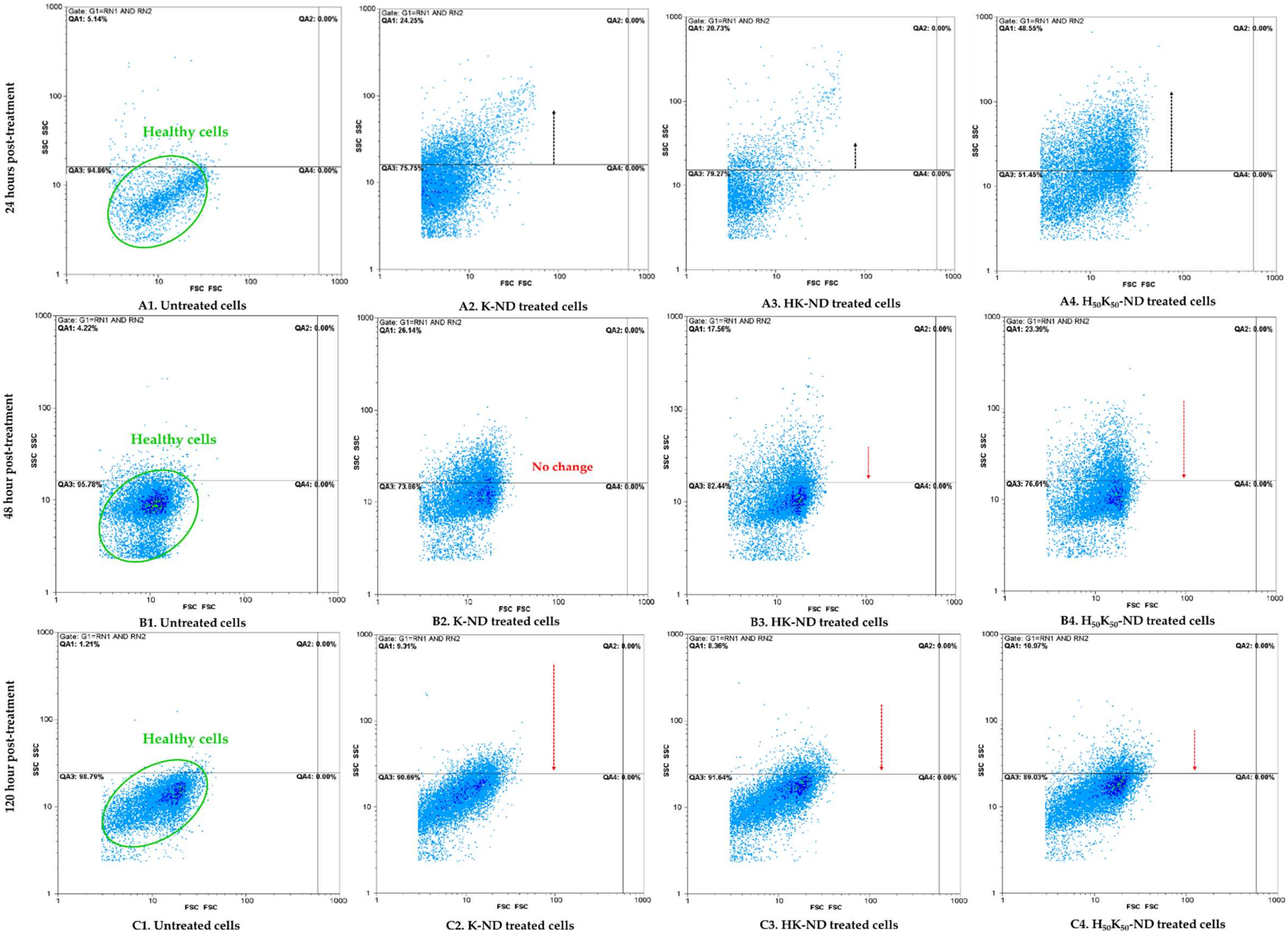

Figure S2. Flow cytometric evaluations of cells treated with various types of NDs and analyzed at three time points i.e., 24-, 48- and 120-hours post treatment. Each graph represents a dot plot of forward scatter (FSC) as the x-axis and side scatter (SSC) as y-axis. Graphs are divided into 4 quadrants to quantify the shift in FSC and SSC. Shift from left to right i.e., from QA3 to QA4 corresponds to increase in FSC due to increase in cell size, while shift from bottom to top i.e., from QA3 to QA1 corresponds to SSC due to increase in granularity and internal complexity of the cells secondary to uptake of NDs. Higher shift from QA3 to QA1 corresponds higher ND uptake. As time progresses from 24 hours to 120 hours back shift from QA1 to QA3 corresponds decrease in SSC due to exocytosis of internalized diamonds. Black arrows show increase in SSC (i.e., shift from QA3 to QA1) while red arrows show decrease in SSC (i.e., shift from QA1 to QA3). Length of arrows corresponds to intensities of the shift. For all samples 10000 events were counted from healthy cell population highlighted in green circle.

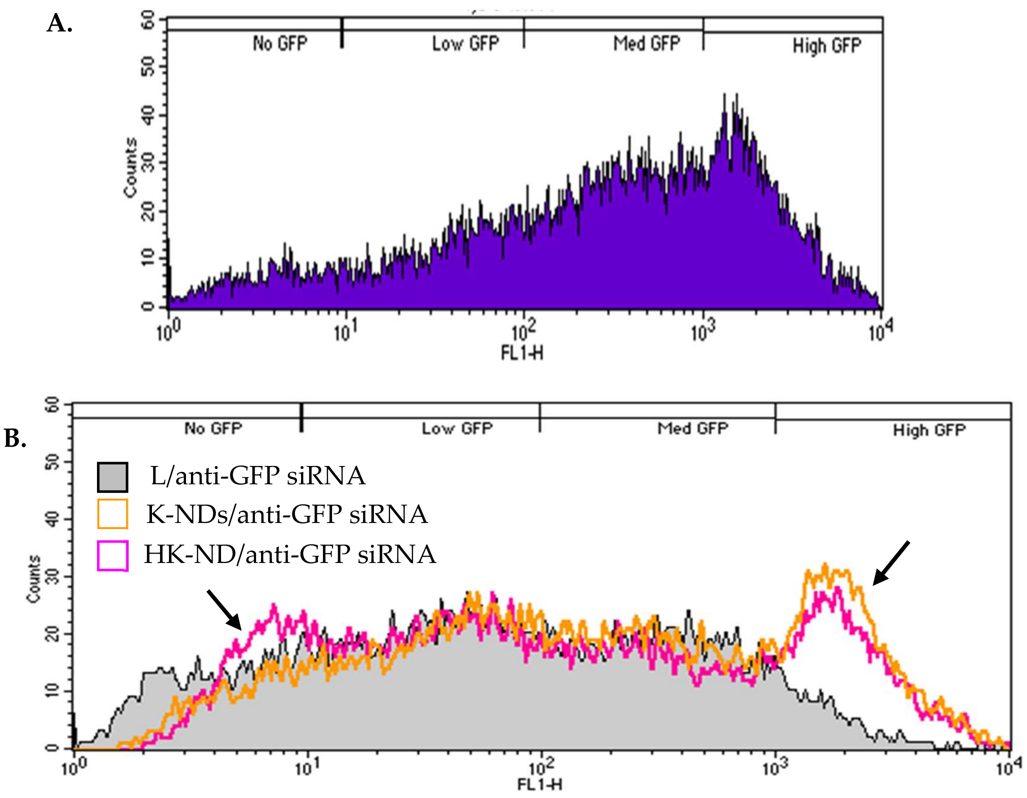

Figure S3. GFP knockdown induced by K-NDs and HK-NDs in comparison with lipofectamine (A) Histogram showing the profile of untreated HeLa-GFP cells skewed to the right i.e., most cells lie in high GFP region and (B) Overlay of histogram comparing GFP knockdown induced by K-NDs and HK-NDs versus lipofectamine after 48 hours of treatment showing that lipofectamine and HK-NDs shows a complete and modest shift from high to no GFP region respectively. *FL1-H on x-axis represents a log scale of GFP fluorescence intensity from analyzed cells from flow cytometry FL1 band pass filter (emission wavelength range = 530 ± 30 nm). Y-axis represent number of cell count in the gated region.
(L= lipofectamine; K-NDs = lysine-NDs and HK-NDs = lysyl-histidine-NDs).

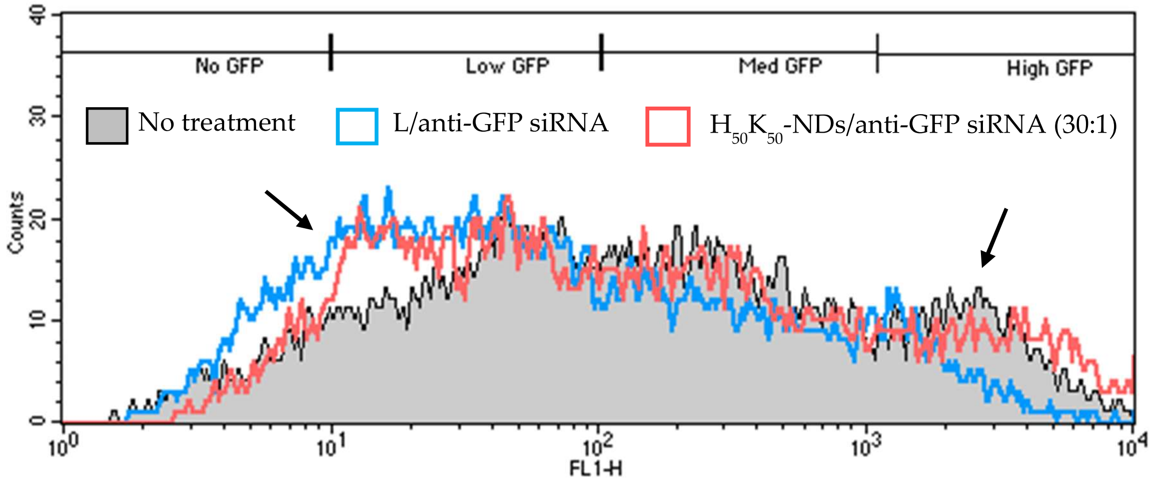

Figure S4. GFP knockdown induced by $H_{50}K_{50}$-NDs in comparison with lipofectamine control and untreated cells. *FL1-H on x-axis represents a log scale of GFP fluorescence intensity from analyzed cells from flow cytometry FL1 band pass filter (emission wavelength range = 530 ± 30 nm). Y-axis represent number of cell count in the gated region. (L= lipofectamine; $H_{50}K_{50}$-NDs = lysine/lysyl-histidine-NDs).

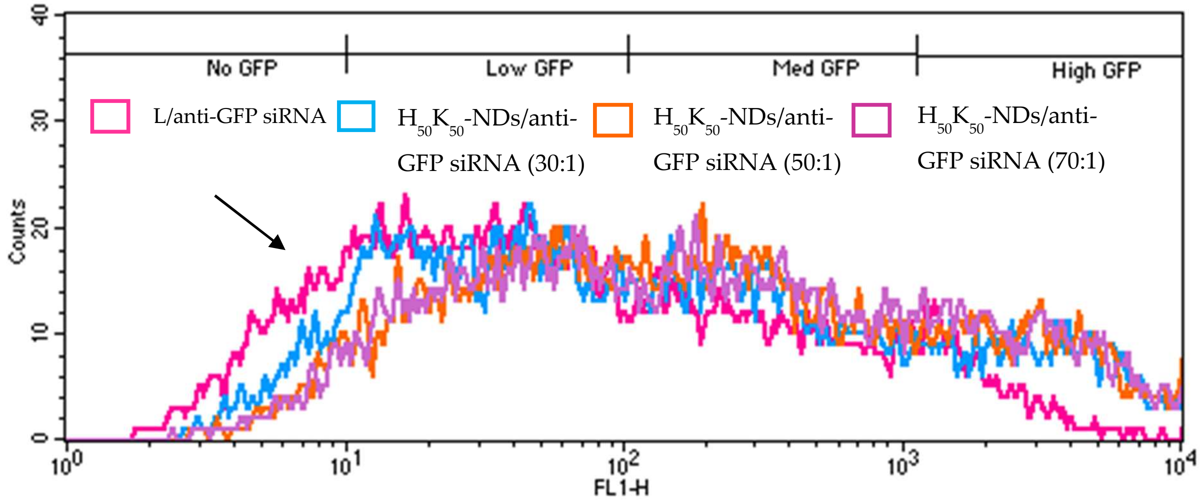

Figure S5. GFP knockdown induced by $H_{50}K_{50}$-NDs at 30:1, 50:1 and 70:1 mass ratio in comparison with lipofectamine control. *FL1-H on x-axis represents a log scale of GFP fluorescence intensity from analyzed cells from flow cytometry FL1 band pass filter (emission wavelength range = 530 ± 30 nm). Y-axis represent number of cell count in the gated region. (L= lipofectamine; $H_{50}K_{50}$-NDs = lysine/lysyl-histidine-NDs).
